# Workplace bullying as the erosion of recognition: ethnographic insights from a Schutzian perspective

**DOI:** 10.3389/fsoc.2026.1735370

**Published:** 2026-01-30

**Authors:** Ángel Martínez-Hernáez

**Affiliations:** Medical Anthropology Research Center, Universitat Rovira i Virgili, Tarragona, Spain

**Keywords:** mobbing, phenomenology (Alfred Schutz), recognition damage, workplace bullying, workplace harassment

## Abstract

This article proposes to understand workplace bullying as the erosion of recognition from a phenomenological perspective. Drawing on Schutz’s sociology of knowledge, it examines how rumours, silences, and informal labels reorganise the typifications through which colleagues are perceived. Once a worker is reframed as unstable, incompetent, or undesirable, ordinary interactions are filtered through this new categorisation. In Schutzian terms, the victim is expelled from the *umwelt* of reciprocity and relocated into the anonymous sphere of “they,” losing not only credibility but also the community of affect that sustains belonging. The analysis is based on ethnographic material from Spain, including in-depth interviews and participant observation in an association of workers affected by workplace bullying. Findings show that processes of misrecognition are not collateral but central to workplace bullying, and that reputational harm emerges as one of their key manifestations. Minor gestures such as exclusion from information flows, disparaging jokes, or subtle task reassignments accumulate into enduring forms of exclusion. These dynamics chronically reorganise common sense, undermine legitimacy, and often persist beyond the initial conflict, revealing why those affected experience long-lasting damage and why institutional responses frequently reproduce rather than repair harm. Conceptually, the article advances workplace bullying research by opening a Schutzian phenomenological line of inquiry that highlights the intersubjective structures through which harassment becomes effective.

## 1 Introduction

In his pioneering research on workplace bullying, Leymann (1990, 1996a,b, 2022) documented dozens of cases in which routine organisational conflicts escalated into systematic campaigns of exclusion. One of the most striking was the case of Helga, the director of a nursing home in Sweden. Initially recognised for her competence, she gradually became the target of her subordinates’ discontent. What began as minor disagreements soon evolved into insinuations that she was “difficult” and “unstable”. Meetings were convened in which administrators questioned her leadership, reframing institutional problems in terms of her supposed psychological fragility. As these insinuations solidified into an institutional narrative, her colleagues withdrew their support, her authority was eroded, and her credibility collapsed. Ultimately, Helga developed social phobia and was excluded not only from her workplace but also from her professional identity.

Leymann described similar processes in other cases. Hans, a kindergarten teacher, faced hostility after criticising organisational practices. What was initially framed as disagreement was quickly pathologised: colleagues interpreted his persistence as obstinacy or latent instability. Isolated and demoralised, he became *persona non grata* among staff. Elisabeth, a mid-level supervisor, was relocated to a basement office and deprived of tasks. When she protested, her withdrawal and visible distress were reinterpreted as signs of depression rather than legitimate resistance. Bertil, a researcher in a technical institute, was targeted by a superior who orchestrated subtle yet persistent attacks on his credibility. Each attempt to defend himself was reframed as a paranoid overreaction. These trajectories illustrate how pathologising responses consolidate as institutional narratives that erode authority and credibility, transforming perception into typification.

For Leymann, these were not anecdotal details but central mechanisms of mobbing, the term he introduced to describe sustained workplace harassment. His insight remains crucial: workplace bullying is not limited to overt hostility (isolation, excessive control) but also includes symbolic acts that progressively undermine reputation and identity (Leymann, 2022). Once a rumour that someone is “unstable” or “undesirable” begins to circulate, it functions as a powerful social weapon, restructuring the victim’s actions through that prism. In this sense, discrediting talk is not mere gossip, but a social transformation that alters the categories through which colleagues interpret one another. The more an identity is othered and pathologised, the clearer it becomes that one is facing systematic harassment.

The central thesis of this article is that reputational damage must be understood as a core mechanism of workplace bullying. Rather than prioritising typological distinctions, this analysis highlights the erosion of recognition as a transversal process that operates across different configurations of power. To conceptualise this, I draw on Alfred Schutz’s phenomenological sociology, which emphasises how social life rests on typifications: shared interpretive categories that allow individuals to make sense of others’ behaviour within a common stock of knowledge (Schutz, 1962, 1967, 1970; Schutz and Luckmann, 1973). In the workplace, such typifications underpin the fragile trust necessary for cooperation. From this perspective, what workplace bullying most profoundly undermines is the mutual recognition that underpins communicative agency and intersubjective reciprocity.

This article is primarily theoretical and conceptually driven, while grounded in ethnographic material. The argument unfolds in several stages. First, I review the foundational and recent contributions to bullying research. Second, I develop Schutz’s perspective in dialogue with theories of recognition. Third, I draw on ethnographic material to illustrate how these dynamics evolve in practice. Finally, I discuss the implications for future research on workplace bullying from a phenomenological standpoint.

## 2 Theoretical framework

### 2.1 Recognition damage and workplace bullying

Leymann (1990, 1996a) defined workplace bullying as a form of violence in organisational settings that generates powerlessness in the targeted person and becomes structural through a chain of hostile acts repeated over time, typically for at least six months. A distinctive feature of this process is that most hostile acts are not overtly aggressive but implicit, indirect, or symbolically mediated. Leymann’s definition was inspired both by Konrad Lorenz’s ethological studies of group harassment among animals and by Heinemann’s work on school bullying, in which groups of students torment a peer to the point of suicide (Leymann, 2022). These influences situated workplace bullying within a broader family of collective violence, characterised by progressive group convergence against a single individual (Westhues, 2011; Hutchinson, 2013; Girard, 1986). From its inception, therefore, the concept pointed beyond interpersonal conflict toward processes of collective alignment and exclusion.

Leymann’s contribution was not only conceptual clarity but also empirical and clinical depth. In Sweden, he contributed to the development of workplace anti-bullying policies, created a specialised clinic that treated nearly 1,400 victims, and developed the *Leymann Inventory of Psychological Terror* (LIPT). This instrument identified forty-five hostile acts, later expanded to sixty by González de Rivera y Revuelta and Rodríguez-Abuín (2003, 2006), grouped into five categories: attacks on reputation and personal dignity, attacks on communication, attacks on social contact, attacks on professional standing, and attacks on health.

On this basis, a vast body of research has framed workplace bullying as a major public health and organisational issue (Hirigoyen, 2016, 2017; Piñuel, 2001; Leymann, 2022; Salin and Hoel, 2011; Nielsen et al., 2024; Chan-Mok et al., 2014). Empirical studies and meta-analyses document its severe consequences for health, including depression, anxiety, burnout, and post-traumatic stress disorder, as well as its effects on performance, absenteeism, and organisational climate (Einarsen et al., 2003, 2011, 2020; Nielsen et al., 2024; Nielsen and Einarsen, 2018; Zapf, 1999, 2004; Zapf et al., 2002; Krishna et al., 2023; Hoel, 2013; Hoel and Cooper, 2000). At an organisational level, these findings have been decisive in legitimising workplace bullying as an occupational hazard requiring prevention and intervention, including the development of taxonomies and decision-making frameworks to guide organisational responses (Caponecchia et al., 2020).

Clinical research has further highlighted the risk of misdiagnosis, whereby victims’ detailed accounts of harassment are interpreted as symptoms of depression, neuroticism, delusional ideas, or personality disorder rather than as responses to sustained social harm (Leymann, 1990, 1996a,b; Martínez-Hernáez and Medeiros-Ferreira, 2010; Nielsen et al., 2012; Nielsen and Einarsen, 2012; Einarsen and Nielsen, 2015; Keashly, 2021). Recent systematic evidence also shows that the psychosocial impact of workplace bullying extends to witnesses, who may experience adverse mental health and work-related outcomes depending on their proximity to the target, identification with them, and the organisational handling of the situation (Nielsen et al., 2024). At the same time, scholars warn against reversed causality, noting how organisations often misread distress as evidence of pre-existing vulnerability rather than as the outcome of social harm (Nielsen and Einarsen, 2012).

Within this literature, workplace bullying has also been classified according to the configuration of power relations involved. A common distinction differentiates downward bullying, in which superiors target subordinates; upward bullying, where subordinates harass a superior; and horizontal bullying, occurring among peers at similar hierarchical levels (Einarsen et al., 2011; Salin and Hoel, 2011; Piñuel, 2001). While analytically useful, these typologies primarily describe formal positions rather than the intersubjective dynamics through which bullying consolidates over time. Empirical research shows that cases often evolve across configurations, as initial horizontal hostility becomes legitimised by management, or downward pressure is reinforced through peer alignment (Hirigoyen, 2016, 2017; Einarsen et al., 2011). This variability suggests that the core mechanism of workplace bullying cannot be reduced to hierarchical position alone.

In parallel, bibliometric analyses show that research on workplace bullying has expanded rapidly over the past three decades, with Italy, Turkey, and Spain among the most productive contexts (Alper Ay, 2025). Four dominant lines of inquiry can be identified: (1) antecedents such as power asymmetries, workload, and organisational culture; (2) perpetrators’ behaviours, particularly vertical bullying through abusive supervision, incivility, and unethical communication; (3) the vulnerability of occupational groups such as nurses, civil servants, teachers, and academics, where horizontal bullying is increasingly prevalent; and (4) the health consequences of bullying, including stress, burnout, and long-term psychosomatic effects. Across these strands, gender, diversity, and cultural factors consistently emerge as risk factors, while bystander inaction remains a critical gap in both research and intervention.

Beyond formal power configurations, research shows that the consolidation of bullying depends on what becomes locally readable as deviance within specific organisational fields. While the underlying phenomenological mechanism recurs, its concrete triggers and modes of visibility vary with what is at stake in each setting. In health care, technical mistakes are readily reinterpreted as incompetence; in education, authority is undermined by insinuations of instability; and in academia, collegial reputation becomes decisive for credibility (Carnero et al., 2008; Milutinović et al., 2009; Reguera and García-Izquierdo, 2021; Zawadzki and Jensen, 2020; Hamzaoglu et al., 2022; Yildirim and Yildirim, 2007; Scaramuzzino, 2024). Gender, age, and migrant status further shape how labels gain traction and how rapidly they become naturalised within common sense and organisational memory (Dassisti et al., 2020). These patterns illustrate that intersectional categories preload workplace interpretive frameworks with what may be termed “credible discredit” (Crenshaw, 1989; Collins and Bilge, 2020), rendering certain bodies more readily legible as unstable or incompetent.

However, while this literature convincingly documents outcomes, risk factors, and health consequences, the intersubjective dynamics through which bullying consolidates have been comparatively less theorised, despite some notable exceptions (e.g., Neuman and Baron, 2010; Faldetta and Gervasi, 2024). What remains insufficiently specified is how hostile acts acquire social force as common sense in everyday organisational life: how rumours, insinuations, and informal labels sediment into shared interpretive frames that reorganise the lived world of work. This analytical gap becomes particularly salient in contemporary workplaces, where digital communication, remote collaboration, and post-pandemic transformations have expanded the spaces in which reputational damage can circulate and persist (West, 2021; Pothuganti, 2024; Eurofound, 2024; Oguz et al., 2023; Aboobaker and Shanujas, 2025; Yan et al., 2025). Yet this gap is not new; it reflects a longer-standing under-theorisation of the phenomenological processes through which misrecognition becomes intelligible, durable, and legitimate.

Several theoretical traditions have moved closer to this problem by foregrounding collective and symbolic dynamics. Scapegoating approaches inspired by Girard (1986) conceptualise workplace bullying as a process of collective alignment in which tensions and conflicts are displaced onto a single target through stereotypical accusations. Recent applications of this framework to organisational settings show how mimetic dynamics can fuel escalating hostility until group cohesion is sedimented through the exclusion of an individual who becomes unable to reciprocate or contest accusations (Faldetta and Gervasi, 2024). Organisational culture perspectives likewise emphasise how shared norms, practices, and tacit rules may normalise bullying and render it morally acceptable or invisible within institutions (Pheko et al., 2017). These perspectives offer crucial insights into collective dynamics. Rather than competing with them, the present article builds on their contributions while addressing a complementary phenomenological analytical level: how, in routine interaction, ordinary encounters come to be reinterpreted and sedimented as common sense.

Leymann’s own work already pointed toward this dimension. His empirical and clinical engagement identified a repertoire of subtle hostile acts, most of them symbolic rather than physical. Like the “moral insult” (Cardoso de Oliveira, 2005), workplace bullying often carries an immaterial appearance that makes it difficult to identify as harm requiring redress, while nevertheless operating as a powerful means of symbolic punishment and expulsion. Rumours, ridicule, silencing, and professional discrediting constitute the core of bullying practices. Taken individually, such acts often appear trivial; taken cumulatively, they reorganise the interpretive frame through which future actions are perceived. Workplace bullying can therefore be understood as a process that unfolds through cascades of meaning. Mundane acts (a sarcastic remark, exclusion from a meeting, a raised eyebrow at the suggestion that someone is “unstable”) gain cumulative force when repeated and shared (Workplace Bullying Institute, 2024). From a phenomenological perspective, repetition habituates suspicion and normalises misrecognition, so that subsequent interactions are pre-shaped by distrust (Salin, 2003; Keashly, 2021).

This dynamic is vividly illustrated in Leymann’s case of Leif, a Danish worker in a Norwegian factory whose accent became the focus of mockery. What began as joking escalated into systematic ridicule and manipulative task assignments that made him appear incompetent: he was repeatedly sent to repair machines that required no repair, earning the nickname “the crazy Dane.” As the jokes spread, his social ties fractured, managers interpreted his frustration as evidence of instability, and workplace stories crystallised into records of poor performance. Over time, anxiety and psychosomatic illness led to sick leave, demotion, and eventual dismissal. With his medical history following him in job applications, Leif became unemployable, a pariah despite a long record of competence in previous workplaces (Leymann, 1990). Here, reputational damage did not merely accompany bullying; it structured how actions, emotions, and bodies were interpreted.

Recent research converges on this view. Reviews emphasise repetition, isolation, and collective storytelling as core features of bullying dynamics (Einarsen et al., 2020; Nielsen et al., 2024), while studies of digital and hybrid workplaces show how reputational frames now circulate with greater speed and durability (West, 2021; Zhang et al., 2021). Disrespect functions as a misreading of the person that redistributes blame (Heidegren, 2022); organisational narratives displace first-person accounts, producing narrative injustice (Vossler et al., 2024); and bodily consequences such as exhaustion are reinterpreted as signs of instability, exemplifying embodied hermeneutical injustice (Fotaki and Harding, 2017). Affective responses are then recoded as further evidence of deficiency, consolidating stigma through the sedimentation of misrecognition and reinforcing group cohesion (Bolton, 2005; Douglas Creed et al., 2014).

Read in conjunction, these approaches establish workplace bullying as a multifaceted social problem. However, they converge on an unresolved issue: the intersubjective dynamics through which reputational damage becomes effective and exclusion sediments as a taken-for-granted reality. What remains under-analysed is how everyday interactions and shared interpretations reorganise the lifeworld of work, withdrawing recognition and credibility. It is precisely at this analytical level that a phenomenological framework becomes necessary.

### 2.2 Schutz and the phenomenology of recognition damage

Alfred Schutz’s sociology of knowledge, rooted in Husserl’s concept of the *lifeworld*, emphasises that social reality is continuously constituted through everyday interaction. The world of work is not a neutral environment but a “finite province of meaning,” governed by its own tension of consciousness, temporality, and practical logic (Schutz, 1962, 1967, 1970; Wagner, 1983; Kim and Berard, 2009). Within this province, cooperation depends on taken-for-granted assumptions of reliability, competence, and good faith. Schutz called this the “natural attitude*”*: the pre-reflexive confidence that the world and others are as they appear (Schutz, 1967). These assumptions are also the minimal conditions under which relations of mutual recognition can be sustained in everyday work. Although Schutz did not address workplace bullying as such, his conceptual apparatus makes it possible to specify what is disrupted when these assumptions erode. Workplace bullying unsettles precisely this horizon. It interrupts tacit trust. What a Schutzian perspective adds is not another typology of bullying or an alternative causal explanation, but the capacity to specify how meaning itself is reorganised in everyday intersubjective interaction.

At the centre of Schutz’s analysis lies the “stock of knowledge at hand,” the intersubjective reservoir of experiences, typifications, and relevances that makes the world appear already ordered and intelligible (Schutz, 1967, 1970). Through it, individuals know how to read situations and to locate others within familiar categories such as colleague, supervisor, novice, or expert. Such typifications are not merely cognitive shortcuts. They shape expectations of competence, reliability, and worth that underpin recognition. These categories are not neutral: they carry expectations. From this perspective, one can observe how, in situations that later come to be recognised as bullying, a person’s typification is displaced. Someone once regarded as dependable or diligent is redefined as unstable, hypersensitive, or inadequate. The change is not only semantic but affects the person’s social being within the workplace. Schutz termed “because motives” (“she insists because she cares about accuracy”) are reinterpreted as “in-order-to motives” (“she insists in order to obstruct or control”) (Schutz, 1967, pp. 95–101). The same act acquires a new intentionality.

Typifications do not exist in abstraction; they are enacted and confirmed through repetition. Each encounter (a look, a delayed reply, an exclusion from informal talk) reinforces the altered reading. As experiences accumulate, they sediment into what Schutz called “habituality,” the inertia of the familiar (Schutz, 1962, pp. 229–231). Once routinised, the reinterpretation no longer requires active justification. Common sense silently reorients its “relevance structures” (Schutz, 1970, pp. 74–76). At this stage, recognition is no longer actively withdrawn but tacitly presupposed as unwarranted. Through this process, the practical evidence of trust erodes: the background of collegiality that sustained recognition is gradually replaced by suspicion.

The phenomenological architecture of this shift is illuminated by Schutz’s distinction between the *umwelt* and the realm of “they” (Schutz, 1967). In the *umwelt*, others are encountered as “you,” co-subjects with whom one shares projects, time, and affective proximity. In the realm of “they,” by contrast, others are perceived only through typifications, as anonymous cases or roles. From this perspective, situations that come to be recognised as bullying can be understood as involving a transition from the *umwelt* to the “they”: the target is expelled from reciprocity and becomes an object of categorisation. Presence in meetings, corridors, or emails no longer counts as participation but as a problem to be managed. The targeted person’s perspective ceases to shape collective sense-making. This loss of reciprocity also distorts what Schutz called the “reciprocity of perspectives,” the everyday assumption that others see the world in roughly similar ways (Schutz, 1967). Cooperation presupposes this symmetry: it allows people to coordinate their intentions and to correct misunderstandings. As typifications shift, this symmetry collapses. Because typifications are collectively sustained, the individual’s self-description loses validity. From this perspective, one can infer that attempts at self-defence may generate a self-reinforcing loop, in which efforts to contest discredit are increasingly read as further evidence of the altered typification, a dynamic akin to what Ian Hacking has described as “looping effects” between classifications and those classified (Hacking, 1995).

Temporality is central to Schutz’s account of social experience and provides a useful entry point for approaching workplace bullying phenomenologically. Bullying does not unfold through isolated events but through temporally extended configurations in which each episode reorients the horizon of expectation. Schutz’s concept of *sedimentation* captures this gradual process: meanings congeal through repetition until they become taken for granted within everyday understanding (Schutz, 1962). In organisational settings, such sedimentation often acquires material form in minutes, reports, emails, or informal records that preserve and circulate altered typifications. What may begin as a tentative reinterpretation of a colleague’s conduct can thus become sedimented through documentation, acquiring what Schutz and Luckmann (1973) describe as a form of “ontological solidity.” Over time, organisational memory may retrospectively reorder biographies, reclassifying past actions and dispositions in light of the new interpretive frame (Schutz, 1970).

From this phenomenological standpoint, workplace bullying can be approached not only as a disturbance of interpersonal relations but as a transformation in the intersubjective conditions under which actions are rendered intelligible. As typifications shift and sediment, the background assumptions that sustain predictability and mutual orientation in everyday work are altered. Reputational damage, in this sense, is not reducible to individual perception or psychological impact; it involves changes in shared meaning structures through which belonging and participation are ordinarily sustained. These dynamics resonate with questions developed in adjacent theoretical traditions concerned with recognition (Honneth, 1996, 2010, 2014; Voswinkel, 2012; Deranty, 2008; Schweiger, 2025), stigma (Goffman, 1963; Link and Phelan, 2001; Yang et al., 2007), and credibility (Fricker, 2007; Dotson, 2014). While these traditions address the moral, symbolic, or epistemic consequences of exclusion and discredit from different theoretical standpoints, they do not specify the phenomenological processes through which altered typifications reorganise everyday interaction.

## 3 Methodology

### 3.1 Research design and context

The ethnographic material analysed in this article was generated during long-term research on workplace bullying in Spain. Fieldwork was conducted between 2002 and 2010 in an “anti-mobbing” association based in Barcelona and formed part of a broader project on the psychosocial consequences of workplace harassment. The research included in-depth interviews, participant observation and informal conversations with workers affected by bullying, as well as with professionals involved in clinical and organisational responses. Findings from this earlier study were previously discussed in a separate publication (Martínez-Hernáez and Medeiros-Ferreira, 2010) that examined diagnostic and clinical misinterpretations of bullying in mental health care. The present article revisits the same corpus from a different analytical perspective, centred on phenomenology, recognition, and intersubjective meaning-making. The aim is not to provide new empirical results but to illustrate the phenomenological viability of Schutz’s framework and to demonstrate how the erosion of recognition can be observed in the lived texture of social experience.

While the data were collected before the widespread digitalisation of work and before the adoption of the *International Labour Organization’s Convention No. 190 on Violence and Harassment* (International Labour Organization, 2019), the phenomenological logic of the processes described has not changed. The forms of communication and organisation may have evolved, yet the underlying processes of reputational erosion remain structurally and experientially continuous. The older ethnographic material thus retains analytical validity for examining the intersubjective dynamics through which recognition is eroded in contemporary workplaces.

### 3.2 Participants

The study comprised twenty-two participants, seventeen of whom identified themselves as victims of workplace bullying and five as professionals directly involved in clinical or organisational intervention: two psychiatrists, two psychologists, and one mental health nurse. Among the victims, six were interviewed twice, four on three occasions, and two on four occasions, providing longitudinal depth to the data. The professionals, by contrast, were each interviewed once. Participants were recruited through support groups within the association. Inclusion criteria required self-identification as a target of sustained bullying at work, while professionals were selected for their experience in therapeutic treatment or institutional response. Participants included women and men working across both public and private organisations. Table 1 summarises the main characteristics of the seventeen participants who reported experiences of workplace bullying.

**Table 1:** Characteristics of participants reporting experiences of workplace bullying.

Category	Subcategory	*n*
Age	20–35	5
	35–50	4
	>50	8
Gender	Women	11
	Men	6
Employment status	Permanent	10
	Temporary/fixed-term	7
Position in organisation	Non-managerial	6
	Middle management	7
	Senior/supervisory	4
Sector	Health care	3
	Education/academia	3
	Public administration	5
	Private enterprises (technical and administrative work)	4
	Construction, services, manual labour	2
Organisation type	Public	10
	Private	7
Predominant bullying configuration	Horizontal	8
	Downward	4
	Upward	5
Interview design	Single interview	5
	Longitudinal (≥ 2 interviews)	12

### 3.3 Data collection and analysis

Data collection combined in-depth interviews with participant observation within the association, documented through a field diary in which observations, interactions, and emerging reflections were systematically recorded. The researcher attended fifteen meetings over several years until the association’s dissolution in 2010, which provided a longitudinal perspective on collective narratives and shared interpretations. Interviews were open-ended and encouraged participants to recount their experiences in their own terms.

The analysis followed a phenomenological and hermeneutic orientation consistent with an abductive logic, focusing on meaning structures, experiential textures, and interpretive shifts rather than on behavioural indicators or variable-based patterns. Each narrative was treated as a lived world and analysed in relation to its biographical and organisational context. Interpretations were refined through iterative reading, triangulating between interviews, observations and contextual materials with analytical emphases shifting over time in response to tensions, inconsistencies, and recurrent questions arising from the material itself. In this abductive sense, theoretical concepts were mobilised to account for empirical ambiguities and were progressively refined in dialogue with the narratives rather than applied as *a priori* explanatory schemes (Martínez-Hernáez, 2014; Tavory and Timmermans, 2022).

Although no formal coding system was applied, recurrent motifs, such as silencing, displacement, irony, and the withdrawal of reciprocity, were identified and discussed in analytic memos. These motifs were not treated as fixed categories but as provisional interpretive condensations, guiding further engagement with the narratives and remaining open to revision as analysis progressed. The process combined analytic memo writing with systematic reflection on emerging interpretations, ensuring coherence while remaining faithful to participants’ experiential language. Specifically, the present article reuses these anonymised materials to conduct a secondary interpretation framed by Schutz’s phenomenology of the lifeworld and theories of recognition. In line with an abductive approach, Schutzian concepts are introduced where they illuminate otherwise opaque aspects of the material, rather than serving as a classificatory grid imposed on the data.

### 3.4 Ethical considerations

All participants provided verbal informed consent after being informed of the aims of the research, the voluntary nature of their participation, and their right to withdraw at any time without consequences. Verbal rather than written consent was chosen because several participants expressed concern that signing documents might expose them to further risk within their workplaces. Participants were also informed that anonymised data could be used for future scholarly analyses related to workplace environments and psychosocial risks.

The research was conducted under the ethical oversight of the Universitat Rovira i Virgili, in accordance with the Code of Ethics of the American Anthropological Association (1998) and the Spanish national regulations then in force. At that time, formal ethical review was not required for non-interventional qualitative studies. Although the original fieldwork predated the EU General Data Protection Regulation (2016/679) and the Spanish Organic Law on Data Protection and Digital Rights (3/2018), data management practices anticipated the principles later codified in those frameworks, including informed consent, data minimisation, pseudonymisation and restricted access.

All identifying information was removed or altered. Audio recordings were destroyed after transcription, and pseudonymised transcripts were securely archived under restricted access for potential scholarly reuse in accordance with the conditions of the original informed consent. The present analysis draws on these archival, anonymised materials. No personal identifiers have been retained that could allow the re-identification of participants.

Given the sensitivity of the topic, participants were reminded of their right to interrupt or terminate the interview at any point. No participant reported distress or discomfort during or after the interviews. On the contrary, several participants expressed a sense of relief at being able to narrate and reflect on their experiences in a confidential and respectful setting. No financial incentives were offered. All procedures were carried out in accordance with the Declaration of Helsinki.

## 4 Experiencing recognition damage: ethnographic narratives

Most participants in this study were members of the previously cited “anti-mobbing” association, which rented a modest room in a working-class district of Barcelona, where meetings were held (usually weekly but often fortnightly) with uneven attendance. During the fieldwork period, the association’s activity revolved around three functions: mutual support; legal, medical, and psychological advice; and lobbying public authorities to reform the legal framework in line with emerging guidance from the International Labour Organization and the European Commission, which urged member states to legislate against psychological violence at work (Di Martino et al., 2003). Pressure from associations of this kind contributed to gradual legal and jurisprudential developments in Catalonia and Spain, leading to the recognition of workplace bullying as a psychosocial risk. It was within this setting that participants narrated their experiences.

Whether they reported upward, downward, or horizontal bullying, participants converged on a common feature: the gradual erosion of credibility and recognition. This convergence did not stem from a single decisive episode but became visible through the accumulation of recurrent micro-transformations in workplace life, such as silences, shifts in tone, minor exclusions, or malicious rumours. Mrs. B, a laboratory technician, described the first turning point in these terms:

“One day we ran out of reagents in the laboratory and I protested loudly. How could that be, when we had so many analyses pending? But my complaint found no echo. Nobody paid attention. From that moment everything changed for the worse. I had become a bothersome and undesirable person.”

What stands out in this account is that the action itself was not exceptional. Complaining about missing materials had previously been part of her routine conduct and, apparently, had not attracted negative attention. Yet after this episode, the same behaviour prompted indifference and, soon after, avoidance. Mrs. B no longer participated in the coffee breaks where workers collectively exchanged everyday matters; gradually, she was pushed out of this shared space through jibes and insinuations that made it clear it had become hostile to her presence.

More generally, participants often stressed this discrepancy: they did not feel they had changed their conduct, while the meaning attributed to that conduct had shifted. Initially, this change did not take the form of explicit accusation. Instead, it appeared through disruptions in ordinary cues of collegial recognition: replies stopped, requests went unanswered, and routine coordination began to falter. In Schutzian terms, something had shifted in the stock of knowledge at hand, enabling new readings and typifications.

In most cases, this displacement did not present itself as a recognisable conflict but as a period of interpretive uncertainty. Participants described an initial phase in which the change in their standing was sensed rather than understood, without a clear trigger to which they could respond. Only retrospectively, as similar reactions accumulated, did a pattern become legible. Crucially, this reclassification did not operate through open confrontation but through everyday interactional shifts: non-response, lack of acknowledgement, and the withdrawal of routine cooperation. As these micro-transformations sedimented, the altered reading of the person began to circulate beyond individual encounters. In several accounts, it provided the conditions for more visible and publicly staged forms of exclusion, in which discredit was collectively enacted.

Such escalation is evident in the case of Mrs. T, an administrative worker in an insurance company, who described how, over time, she was repeatedly spat at as she entered her workplace: “Sometimes it was discreet, from a window when they saw me arrive; other times it was plain and direct. Afterwards the aggressor and the onlookers laughed loudly so that I would hear them.” What is salient in this account is its repetition and public character. Spitting was not described as an isolated outburst but as a recurrent, collective gesture. The entrance to the workplace became the staging of a lynching and an expulsion from the *umwelt*.

While the explicit and physical humiliation described by Mrs. T was exceptional, it illuminates a dimension that recurred across narratives: the bodily inscription of discredit. Even when hostility remained largely within the symbolic register, many participants emphasised its corporeal impact. The problem was not only the act itself, but how it was lived through anticipation, exposure, and the impossibility of avoidance, leaving a durable temporal imprint. Some described changing their arrival times, routes, or bodily posture to reduce visibility; others reported taking anxiolytics or other medication to endure the situation. Such strategies rarely altered the dynamics at play. At most, they attenuated immediate exposure, at the cost of reinforcing the sense of being marked, of losing spontaneity, and of having to regulate one’s body differently from co-workers. What began as a change in how colleagues or managers read the person thus consolidated into a shared script in which the individual was treated as legitimately degradable. The repetition of these gestures did not merely express hostility; it sedimented it.

Participants consistently emphasised the implicit and insidious character of hostility. In their narratives, several noted that explicit aggression, however distressing, could be clarifying, in contrast to indirect allusions that generated doubt and prolonged disorientation. Mrs. D, a mid-to-senior public-sector employee, described how for months she tried to disregard veiled remarks and repeatedly questioned whether she was being overly suspicious of her colleagues. During this period, the problem remained suspended at the level of interpretation. The ambiguity eventually crystallised into an administrative decision. With little explanation from management, she was transferred to another office with no defined function, a move that amounted to a clear downgrading of her occupational standing. From holding responsibility for auditing public-works expenditure across several municipalities, she was relocated to a basement office with no concrete tasks.

As in Mrs. D’s case, most accounts showed exclusion taking shape through practices whose meaning remained ambiguous and whose effects only became legible over time. Sometimes, exclusion was enacted through everyday interactions that appeared benign at first. Mrs. J, a municipal employee who later took early retirement, described how ordinary sociability became a vehicle for discredit:

“Sometimes someone asked you kindly about your life and you, naively, told them everyday things, about your family or the weekend, because you want to have a good relationship. Then you realise they asked you only to have material for gossip.”

In this account, routine exchanges were retrospectively reinterpreted as strategic. The participant did not describe an immediate sense of threat, but a delayed recognition, once fragments of her private life began to circulate in distorted form at work. When she took medical leave for gallbladder surgery, for example, this absence was reclassified by the bullying group as a psychiatric admission linked to her supposed psychological instability. What had initially functioned as ordinary sociability was thus reworked into a mechanism of reputational erosion.

A related dynamic appeared in accounts centred on silence and informational exclusion. Mrs. H, an administrative worker in a service company, recalled:

“The first sign things were going wrong was silence. Suddenly information stopped reaching me. I discovered decisions had been taken without me, despite my responsibilities. They did not inform me, they ignored me. They made me look irresponsible. Soon this turned into gossip, blaming me for not being informed, and I was left looking foolish. If you tell them they are pissing on you and expect them to say it is raining, they will also treat you as paranoid.”

Here, isolation and silence initially replaced any explicit accusation. At first, the participant oscillated between organisational explanations, misunderstanding, and self-doubt. Only later did she grasp that the absence of information had itself become the basis for attributing irresponsibility. Over time, what appeared as a mundane organisational malfunction was transformed into shared common sense, and a new, discrediting account of her competence was sedimented.

Participants often described an initial phase of confusion. Something had shifted, yet there was no clear language with which to name it. This uncertainty delayed response and made resistance difficult. The situation could be denied or reframed as coincidence, misunderstanding, or personal sensitivity. Over time, however, the accumulation of such episodes produced a growing sense of social disappearance. Several participants resorted to metaphors of erosion or emptiness to describe this experience. Mr. A, a senior civil servant at an upper-middle level, articulated this condition through a striking image:

“At first you try to rebuild relationships [at work]. You think that if you explain yourself, if you make an effort, things can go back to normal. Then you realise that it only means exposing yourself again and again, and that it is not worth it. They see you like a zombie, like someone who is half dead and half alive, like a corpse that has returned from the underworld. That gives them permission to do things they would not do to someone else, like spreading rumours, making up your private life, or removing documents from your office. They feel protected by the group.”

This account captured a condition repeatedly evoked across narratives. What was described was not a conflict with specific colleagues but a transformation in social address: being spoken about rather than spoken with. Participants portrayed themselves as inhabiting a social limbo, physically present yet no longer recognised as fully legitimate interlocutors. The zombie metaphor condensed this experience of suspended social life and the withdrawal of recognition. Once the person came to be tacitly re-typified in this way, actions such as collective mockery or the circulation of rumours, which would normally trigger restraint, became permissible. Processes of reputational damage thus emerged as the cumulative reworking of everyday interaction through which gossip, silence, and selective communication sedimented a shared understanding of the person as unreliable, problematic, or deficient.

Several participants described this process in terms of experiential estrangement. While their sense of self remained coherent outside work, within the organisational setting they encountered a growing dissonance between how they understood themselves and how they were addressed by others. Participants often spoke of feeling like different versions of themselves across contexts: recognised and credible in family life, friendships, community engagement, or even other work settings, yet persistently questioned, diminished, or rendered suspect at work. From a Schutzian perspective, this experience reflects a breakdown in the continuity between finite provinces of meaning. Recognition available in one social world no longer travelled into the lifeworld of work, where altered typifications prevailed. This discontinuity intensified feelings of unreality and injustice, as participants struggled to inhabit everyday worlds governed by incompatible expectations and interpretive frames.

For most participants, attempts at response or resistance emerged once exclusion became unmistakable. These responses were diverse and often improvised, ranging from ironic self-labelling to open confrontation or strategic withdrawal. Mr. L, a middle manager, described how he sought to anticipate and neutralise insults by appropriating the label imposed on him:

“I know my colleagues see me as mad, so I usually tell them: ‘Careful, I’m a dangerous lunatic.’ Sometimes, when I see them waiting with their smiles to launch a group insult, I smile and say: ‘How happy you look, how nice,’ and then I see their faces turn serious with guilt, and I laugh.”

Such strategies were described as attempts to recover a minimal sense of agency in an environment where interactions were already anticipated and scripted by others. Yet participants consistently emphasised that these responses did not disrupt the underlying intersubjective dynamic. Irony, humour, or open defiance were quickly reabsorbed into the existing narrative, becoming further confirmation of the very label being resisted. As the situation unfolded, the possibility of restoring reciprocity came to be foreclosed. Several participants reflected on this paradox with frustration. Efforts to clarify misunderstandings or defend professional competence tended to intensify rather than interrupt hostility. Emotional reactions were equally ambivalent: expressing anger, distress, or exhaustion risked reinforcing the image of instability, while suppressing them deepened isolation. As Mrs. MJ, a schoolteacher, succinctly put it: “Whatever you do, it all fits, because everything has already been made to fit.”

In some cases, the reputational frame exceeded everyday interaction and entered clinical or bureaucratic domains. Mrs. MA, a nurse, recalled how, after months of exclusion and rising anxiety, she sought medical help: “I told the doctor everything, step by step, together with my husband. I had it all written down and ready. The response was: ‘It’s all in your head.’” For her, this moment marked a qualitative shift. Interpretations that had circulated informally among colleagues were now reproduced in a clinical setting, acquiring a different authority and durability. Several participants described comparable experiences in which workplace narratives of instability resurfaced in occupational health assessments or administrative procedures. At this point, reputational damage was no longer confined to everyday interaction but became embedded in institutional records and expert judgements.

Other testimonies underscored the public and performative dimension of lay pathologisation. Mr. M, a university lecturer, described how insinuations about his sexuality gradually transformed into veiled accusations of instability. A joke made during a staff committee that included student representatives triggered a process of harassment. It drew them in as accomplices for some academics seeking to displace him from internal competition for a permanent post. Anonymous notes soon appeared under his office door, “always ambiguous enough that, if I reported them, they would have taken me for an unbalanced person overinterpreting a tasteless joke; a trap I did not, of course, fall into.” Despite this, the same colleagues who initiated the aggression claimed behind his back that he suffered from a mental health problem in order to discredit him, an attribution that he only became fully aware of years later. Although his wife, a psychiatrist, later stated clearly that Mr. M did not have a mental disorder, her medically informed account did not interrupt the ongoing harassment. The episode illustrates how narratives of pathologisation can persist and solidify independently of factual evidence when what is at stake is the symbolic expulsion of a person from the *umwelt* of collegial interaction.

Mr. G, a construction worker with a mild stutter, described a different but related trajectory of collective targeting: “Every morning my colleagues, including the foreman and the owner, greeted me together: ‘G-g-g-good morning.’ The more they mocked me, the more I stuttered.” Here, mockery did not simply reflect a supposed deficiency; it actively intensified it. Several participants pointed to this circularity, noting how reactions produced by harassment were later cited as proof of delegitimation. What was initially reactive and situational came to appear fixed and inherent, distorting identity and eroding recognition.

Across narratives, a recurrent pattern emerged. Once a negative reading had consolidated, it tended to organise subsequent interactions across contexts. Attempts at resistance, explanation, or withdrawal did not reset interpretation but were absorbed into the shared order of meanings already established by the bullying group. Participants often described feeling trapped in a loop. At this stage, several clinicians interviewed for the study stressed the difficulty of reversing such processes. Dr. Z, a psychiatrist, explained:

“They arrive disorientated and confused, unable to see that they cannot change things, and unaware that their managers and colleagues will continue to think and act in ways that serve their own interests. They are in a toxic environment and struggle to recognise it. There is often a naïve belief that things can be fixed, especially when earlier relationships were good, and I have to tell them: ‘Do you see where we are? You’ve come to a psychiatrist because of this. Does that tell you anything? Find another job and forget that nightmare.’ […] I have seen many people harmed by this kind of violence; losing a job is painful, but when they do leave, you see how they recover. They seem like different people.”

For those affected, leaving was rarely experienced as a neutral solution. Some saw it as capitulation; a confirmation of the narrative that had been imposed on them. Others described it as the only way of escaping a situation in which their presence had become socially untenable. In both cases, departure did not merely mark a change of employment but the end of a form of social belonging that had previously been taken for granted.

Resignation, however, was not always framed as withdrawal. In some accounts, it was explicitly rejected as unacceptable, both morally and materially. Mr. S, a security guard, expressed this position with clarity:

“Resigning? Not a chance. If you resign, everything you’ve suffered up to that point has been for nothing, and on top of that you leave without unemployment benefit and without compensation. What you have to do is record the aggressions. An audio recording on your phone is enough. Then you file a complaint.”

Here, resistance and documentation were framed as counterstrategies aimed at restoring a minimal sense of justice or recognition through legal means. Recording and filing complaints were described as ways of interrupting the existing interpretive asymmetry, forcing what had circulated informally to become publicly accountable. At the same time, this stance illustrates the costs of staying. Persistence required vigilance, self-monitoring, and constant anticipation of further aggression, turning everyday work into a space of continuous alertness.

Mrs. MC narrated a comparable impasse, highlighting the silence of workplace “observers”:

“After 12 years in the company, I went on sick leave for depression. My GP advised me to seek legal counsel. If I file a claim for psychological and sexual harassment, what chances do I have? No colleague is willing to testify; they do not want to risk their job and say they have not seen anything directly. […] Only one former employee has agreed to testify. I have no proof.”

Considered together, these divergent orientations towards leaving or staying expressed the same dilemma. Whether through resignation, resistance, or legal confrontation, participants described themselves as operating within a narrow horizon of possibilities. What was lost was not only a job or a position, but recognition as a legitimate participant in a shared world of work. The harm exceeded emotional distress or dissatisfaction. Participants described a deeper alteration: remaining organisationally present while becoming relationally absent, expelled from the logics of reciprocity and trust that ordinarily sustain workplace life.

From an ethnographic perspective, these narratives point to a transformation that cannot be adequately captured by focusing solely on overt acts, individual vulnerability, or formal organisational structures. What becomes visible instead is a gradual reorganisation of everyday common sense, through which people are reclassified, interactions lose reciprocity, and exclusion comes to appear reasonable or inevitable. It is this experiential texture, marked by the erosion of recognition, that calls for a phenomenological interpretation.

## 5 Discussion

Read through Schutz’s phenomenology of the lifeworld, the ethnographic cases examined here show how sequences of hostile acts acquire social force through changes in shared interpretive frameworks. Across narratives, the ordinary conduct of targeted individuals comes to be progressively reinterpreted through frames of discredit that gain plausibility within the shared stock of knowledge of the bullying group (Schutz, 1967). As these frames consolidate, hostility no longer depends on the severity of individual acts but on the intersubjective reorganisation of meaning through which actions are anticipated, read, and responded to. These interpretive schemes operate largely at a pre-reflexive level, reshaping common sense and altering the reciprocity of perspectives that sustains everyday cooperation. Over time, this reorientation expels the targeted worker from social belonging and from the community of affects that underpins collegial interaction. Participants repeatedly described this displacement as a movement from being encountered as a “you” within the *umwelt* of shared projects and proximity to being positioned as a “they,” an object of categorisation rather than a participant in collective sense-making. In this way, reclassification itself comes to legitimise hostility.

A central feature of this process is its temporal structure. Exclusion rarely emerged through a single decisive rupture but unfolded through the accumulation of minor interactional shifts: unanswered messages, delayed replies, silences in meetings, or the gradual withdrawal of routine cooperation. Taken individually, such gestures appeared trivial or ambiguous. In combination, they produced a durable alteration in how the person was read and addressed. In Schutzian terms, these experiences illustrate how typifications sediment through repetition until they acquire the status of institutional facts (Schutz and Luckmann, 1973). As encounters accumulated, the altered reading of the person no longer required explicit justification. Relevance structures silently reoriented, and recognition ceased to be actively withdrawn because it was tacitly presupposed as unwarranted. Workers remained physically present in organisational spaces but increasingly absent from the intersubjective order that sustains cooperation. Table 2 synthesises these recurrent intersubjective dynamics, linking ethnographic cues to shifts in typification, reciprocity, and relevance within the lifeworld of work, and to their effects on recognition.

**Table 2:** Recurrent intersubjective dynamics in the erosion of recognition at work (a Schutzian reading).

Ethnographic cues	Intersubjective reorganisation	Schutzian analytical dimensions	Effect on recognition
Silence, unanswered messages, delayed replies	Withdrawal of reciprocity and disruption of taken-for-granted coordination	Reciprocity of perspectives; natural attitude	Loss of credibility as a legitimate interlocutor
Ordinary actions reinterpreted as disruptive or problematic	Displacement of typifications governing competence and intent	Stock of knowledge at hand; typification	De-legitimation of professional competence
Being spoken about rather than spoken with	Shift from *umwelt* (“you”) to anonymous categorisation (“they”)	*Umwelt* vs. realm of “they”	Objectification and loss of addressability
Accumulation of minor interactional slights	Sedimentation of misrecognition through repetition	Sedimentation; habituality	Normalisation of exclusion as common sense
Gossip and selective circulation of information	Reorganisation of relevance structures and shared interpretive frames	Relevance structures	Erosion of moral esteem and trustworthiness
Emotional or bodily reactions cited as evidence of instability	Reversal of because-motives into in-order-to motives	Motives (“because”/“in-order-to”)	Moral devaluation and pathologisation
Exclusion from informal coordination and decision-making	Loss of access to the shared interpretive world of work	Finite province of meaning	Withdrawal of recognition as participant in cooperation
Informal discredit reproduced in clinical or bureaucratic settings	Institutional consolidation of altered typifications	Objectivation; ontological solidity	Durable institutionalisation of misrecognition

The ethnographic material further shows that reputational displacement rarely remained confined to everyday workplace interaction. In several cases, interpretations that initially circulated informally among colleagues migrated into clinical, bureaucratic, or administrative domains, where they acquired greater durability and authority. Narratives of instability were reproduced in occupational health assessments, medical consultations, or evaluative procedures, amplifying their effects. Fricker’s (2007) notion of epistemic injustice helps to specify this transition. Participants described both hermeneutical injustice, in the absence of a legitimised semantic register to make sense of what was happening in the early phases of bullying, and testimonial injustice, as their accounts lost credibility in institutional encounters. When a physician dismissed Mrs. MA’s detailed chronology of harassment as delusional, an interpretation forged in the workplace was effectively ratified in a clinical setting. What had been an informal discrediting frame thus became institutionally consequential, reshaping the conditions under which the person could be heard and believed.

Read through the ethnographic material, the erosion of recognition can be specified as a compound injury with analytically distinguishable but empirically entangled dimensions. It is phenomenological in its intersubjective constitution, insofar as bullying consolidates through typificatory displacement and the weakening of reciprocity, relocating the targeted worker from the *umwelt* of collegial address to the anonymous realm of “they” (Schutz, 1967). It is epistemic, because this same shift undermines the authority of the person’s word and narrows the shared interpretive resources through which their experience can be rendered intelligible and credible, producing patterned forms of epistemic injustice and epistemic oppression (Fricker, 2007; Dotson, 2014; Byskov, 2020; Medina, 2013). And it is moral, because the withdrawal of social esteem corrodes the evaluative conditions under which belonging is sustained over time (Honneth, 1996, 2010, 2014; Schweiger, 2025). Across cases, these dimensions did not appear as separate layers but as mutually reinforcing aspects of a single process unfolding across interactional and institutional settings.

From a Schutzian perspective, stigma and scapegoating are best understood not as sequential stages following typificatory change, but as crystallisations of this erosion of recognition within everyday interpretive practices. As typifications shift, actors’ “because motives” (grounded in past experience) are progressively reinterpreted as “in-order-to motives,” so that ordinary actions come to be read as intentional, strategic, or suspect. As altered readings of the person circulate and sediment, stigmatising categories provide moral shorthand through which exclusion becomes normalised and justified (Goffman, 1963; Yang et al., 2007). In some organisational contexts, scapegoating further concentrates diffuse tensions and uncertainties onto a single figure, rendering symbolic lynching and exclusion naturalised and reproduced through mimetic escalation (Douglas Creed et al., 2014; Faldetta and Gervasi, 2024). What Schutz’s phenomenology makes visible here is not causal priority but the sedimentation of misrecognition as common sense.

This conceptual framing has implications for how workplace bullying is understood at an organisational level. If typifications are the medium through which bullying consolidates, early signs of exclusion are likely to appear not only in overt hostility but in subtle routines that reshape interactional expectations. Selective flows of information, exclusion from informal coordination, or the recoding of previously neutral behaviours as disturbances emerge analytically as warning signs of reputational erosion rather than as neutral organisational malfunctions. Documentation practices also warrant critical attention. Evaluative records, clinical descriptors, or informal notes can function as sites where sedimented biases acquire ontological solidity. Without contextualisation and periodic review, such inscriptions risk transforming the effects of aggression into enduring stigma. These observations do not amount to a prescriptive toolkit but illustrate how phenomenological insight can inform organisational reflexivity by clarifying how exclusion becomes self-reinforcing.

The central contribution of this article lies in showing that workplace bullying cannot be fully understood by focusing only on hostile acts, individual vulnerability, or formal organisational structures. What the ethnographic material brings into view is a gradual reorganisation of everyday common sense, in which recognition is withdrawn, reciprocity erodes, and exclusion comes to appear inevitable. By situating these processes within Schutz’s phenomenology of the lifeworld, the analysis specifies how misrecognition operates as an intersubjective transformation that destabilises cooperation and belonging in working worlds.

## 6 Conclusion

Conceptually, this article introduces Schutzian phenomenology into the study of workplace bullying, a perspective that, to the best of my knowledge, has not previously been systematically developed. By bringing phenomenology into dialogue with hermeneutical, epistemic, and moral theories of injustice it shows how typifications reorganise the lifeworld of work. By treating recognition as the elementary medium of cooperation, it clarifies how seemingly minor acts restructure the stock of knowledge and initiate processes of sedimentation through which exclusion becomes durable and self-evident. The ethnographic cases illustrate how credibility is gradually withdrawn until colleagues are no longer encountered as “you” within the *umwelt* of reciprocity but as “they” within the anonymous realm of classification. This passage from reciprocity to typification highlights the compound nature of recognition damage (phenomenological, epistemic, ethical, and interactional).

By reframing workplace bullying as the erosion of recognition within what Schutz would term the finite province of meaning of work, this article opens a line of inquiry into the phenomenology of psychosocial risk. Such an approach invites future research to examine how organisational practices might interrupt the temporal consolidation of typifications before they sediment into forms of institutional exclusion. Ultimately, what is at stake is not only protection from hostility, harm and exclusion, but the conditions under which persons should be recognised as legitimate participants in the shared world of work.

## Data Availability

The raw data supporting the conclusions of this article will be made available by the authors, without undue reservation.
